# Supplementary material for: HIVprotI: an integrated web based platform for prediction and design of HIV proteins inhibitors
Source: J Cheminform. 2018 Mar 9;10:12. doi: 10.1186/s13321-018-0266-y (PMC5845081; doi:10.1186/s13321-018-0266-y)
Supplement: Supplementary file 2 — Additional file 2. Source code of HIVProtI web server. [file 13321_2018_266_MOESM2_ESM.zip › HIVprotI_Source-code/hivprotI/weka-3-6-6/doc/weka/estimators/package-tree.html]

weka.estimators Class Hierarchy


---


|  |  |  |
| --- | --- | --- |
| **Overview** \| **Package** \| Class \| **Tree** \| **Deprecated** \| **Index** \| **Help** \| **Weka's home** | |  |
| **PREV**   **NEXT** | **FRAMES**    **NO FRAMES**     **All Classes** |  |


---


## Hierarchy For Package weka.estimators

**Package Hierarchies:**: All Packages

---

## Class Hierarchy

- java.lang.Object
  - weka.estimators.**CheckEstimator** (implements weka.core.OptionHandler, weka.core.RevisionHandler)- weka.estimators.**CheckEstimator.AttrTypes** (implements weka.core.RevisionHandler)- weka.estimators.**CheckEstimator.EstTypes** (implements weka.core.RevisionHandler)- weka.estimators.**CheckEstimator.PostProcessor** (implements weka.core.RevisionHandler)- weka.estimators.**DDConditionalEstimator** (implements weka.estimators.ConditionalEstimator)- weka.estimators.**DKConditionalEstimator** (implements weka.estimators.ConditionalEstimator)- weka.estimators.**DNConditionalEstimator** (implements weka.estimators.ConditionalEstimator)- weka.estimators.**Estimator** (implements weka.core.CapabilitiesHandler, java.lang.Cloneable, weka.core.OptionHandler, weka.core.RevisionHandler, java.io.Serializable)
                  - weka.estimators.**DiscreteEstimator** (implements weka.estimators.IncrementalEstimator)- weka.estimators.**KernelEstimator** (implements weka.estimators.IncrementalEstimator)- weka.estimators.**MahalanobisEstimator** (implements weka.estimators.IncrementalEstimator)- weka.estimators.**NormalEstimator** (implements weka.estimators.IncrementalEstimator)- weka.estimators.**PoissonEstimator** (implements weka.estimators.IncrementalEstimator)
                  - weka.estimators.**EstimatorUtils** (implements weka.core.RevisionHandler)- weka.estimators.**KDConditionalEstimator** (implements weka.estimators.ConditionalEstimator)- weka.estimators.**KKConditionalEstimator** (implements weka.estimators.ConditionalEstimator)- weka.estimators.**NDConditionalEstimator** (implements weka.estimators.ConditionalEstimator)- weka.estimators.**NNConditionalEstimator** (implements weka.estimators.ConditionalEstimator)

## Interface Hierarchy

- weka.estimators.**IncrementalEstimator**- weka.core.**RevisionHandler**
    - weka.estimators.**ConditionalEstimator**

---


|  |  |  |
| --- | --- | --- |
| **Overview** \| **Package** \| Class \| **Tree** \| **Deprecated** \| **Index** \| **Help** \| **Weka's home** | |  |
| **PREV**   **NEXT** | **FRAMES**    **NO FRAMES**     **All Classes** |  |


---
